# Establishment of anti-mesothelioma monoclonal antibodies

**DOI:** 10.1186/s13104-016-2128-x

**Published:** 2016-06-24

**Authors:** Natsuko Mizutani, Masaaki Abe, Shuji Matsuoka, Kazunori Kajino, Midori Wakiya, Naomi Ohtsuji, Ryo Hatano, Chikao Morimoto, Okio Hino

**Affiliations:** Department of Pathology, Kyorin University School of Medicine, 6-20-2, Shinkawa, Mitaka-shi, Tokyo 181-8611 Japan; Departments of Pathology and Oncology, Juntendo University School of Medicine, 2-1-1, Hongo, Bunkyo-ku, Tokyo, 113-8421 Japan; Department of Pathology, Tokyo Medical University Hachioji Medical Center, 1163 Tatemachi, Hachioji-shi, Tokyo 193-0998 Japan; Therapy Development and Innovation for Immune Disorders and Cancers, Juntendo University School of Medicine, 2-1-1, Hongo, Bunkyo-ku, Tokyo, 113-8421 Japan

**Keywords:** Monoclonal antibody, Mesothelioma, Diagnosis, Therapy, Method

## Abstract

**Background:**

Mesotheliomas are aggressive, therapy-resistant tumors that are predicted to increase in incidence at least until 2020. The prognosis of patients with mesothelioma is generally poor because they are typically diagnosed at a late stage and their tumors are resistant to current conventional therapies. For these reasons, improved diagnosis and therapy are urgently required. To address these issues, the aim of our research was to develop novel mesothelioma-specific monoclonal antibodies (mAbs) as diagnostic and therapeutic agents.

**Methods:**

To develop anti-mesothelioma mAbs useful for diagnosis and therapy, we repeatedly immunized a BALB/c mouse with viable mesothelioma cells, alternating between those from three mesothelioma cell lines. We hybridized the spleen cells from this immunized mouse with P3U1 myeloma cells. We then screened supernatants harvested from the hybridoma clones by assessing whether they bound to a mesothelioma cell line not used for immunization and altered its morphology. We designed this developmental strategy to reduce the risk of obtaining clonotypic mAbs against a single mesothelioma cell line.

**Results:**

Our newly generated mouse anti-human mAbs immunostained clinical samples of mesotheliomas. One of the newly generated mAbs did not react with any other tumor cell line tested. Two other mAbs significantly inhibited the proliferation of mesothelioma cells.

**Conclusion:**

These newly generated anti-mesothelioma mAbs are potentially useful as diagnostic and therapeutic agents for mesothelioma. Moreover, our novel strategy for establishing antitumor mAbs may facilitate the development of new diagnostic and therapeutic techniques for mesotheliomas and other malignancies.

## Background

Mesothelioma is an aggressive tumor that develops from mesothelial cells, which line the pleural, peritoneal, and pericardial cavities. The development of mesothelioma is usually associated with chronic exposure to asbestos fibers [1, 2]. The worldwide incidence of mesothelioma is increasing and is expected to peak in approximately 2020 because of the long latent period between exposure to asbestos fibers and the appearance of disease [3]. Asbestos continues to be mined in Russia, China, Brazil, Kazakhstan and other countries [4]. Asbestos is still imported and used in brake pads, gaskets, etc., even in the US [4]. Diagnosis based on chest X-ray and computed tomography (CT) findings must be confirmed by cytologic serous effusion examination or biopsy [5–7].

Immunohistochemistry has significantly enhanced the accuracy of cytology, although reaching a diagnosis remains frequently difficult [8–10]. Although many diagnostic procedures are available, no single test unambiguously distinguishes mesothelioma from other carcinomas or even benign from malignant cells. The correct combination of antibodies used to detect positive or negative markers should be employed and a comprehensive assessment of the staining results must be conducted [11].

Mesothelioma is categorized histologically as epithelioid, sarcomatoid, biphasic, and desmoplastic, among others [9]. The histological subtypes help predict prognosis and the choice of treatment. Therefore, it is important to analyze pathological tissue using an appropriate combination of antibodies for diagnosis and classification. Nevertheless, many cases are difficult to diagnosis [12–16].

Although an anti-CD26 mAb is a promising candidate, no effective molecular target for therapy exists [17, 18]. Therefore, the development of novel anti-mesothelioma mAbs may facilitate diagnosis and serve as therapeutic agents. Subsequent to the development of mAbs by Köhler, Milstein, and Jerne in 1975 [19], excessively large number of mAbs were generated as diagnostic [20, 21] or therapeutic reagents [22].

Diagnostic or therapeutic mAbs are now typically generated by immunizing mice with synthetic peptides or target antigens that are purified to some extent [23, 24]. Except for nude or SCID mice, mice reject inoculated live malignant human tumor cells. During the first or second challenge, tumor cells may be primarily killed by NK and CD8 cytotoxic T cells or ingested by macrophages. However, during the course of repeated immunizations, mouse B cells are generated that produce antibodies against the tumor cells. These antibodies probably make a major contribution to the rejection of the tumor cells. This hypothesis served as the rationale for our experiments that were aimed at establishing anti-mesothelioma mAbs. We report here the generation of anti-human mesothelioma mAbs for diagnosis and treatment. This was accomplished by immunizing a mouse with live mesothelioma cell lines. The hybridomas were selected for their ability to produce antibodies that bound to mesothelioma cell lines not used for immunization. Some of these newly established mAbs reacted specifically with mesothelioma cells or inhibited their proliferation.

## Methods

### Mice

Female BALB/c mice (6–8 weeks of age) were purchased from Charles River Japan (Yokohama, Japan) and housed in a specific pathogen-free facility in micro-isolator cages. Animal experiments were conducted following protocols approved by the Animal Care Committee of Juntendo University of Medicine.

### Cells

The human mesothelioma cell lines ACC-MESO-1 and ACC-MESO-4 were purchased from the RIKEN Cell Bank (Ibaraki, Japan), JMN was a kind gift from Dr. Brenda Gerwin and others (NCI-H226, MSTO-211H, NCI-H28 and NCI-H2452) were purchased from the American Type Culture Collection (ATCC) (Manassas, VA). The lung cancer cell lines (A549, PC9, Lu24, and WA-hT), and the HuH-7 (hepatocellular carcinoma), MKN1 (gastric cancer), OVK18 (ovarian cancer), VMRC-RCW (renal cell carcinoma) cell lines were purchased from the RIKEN Cell Bank. The MCF7 (breast cancer), KP3 (pancreatic cancer), and HCT 116 (colon cancer) cell lines were purchased from ATCC. All cells were cultured and maintained in RPMI-1640 medium (GIBCO, Grand Island, NY) supplemented with 10 % heat-inactivated fetal calf serum.

### Antibodies

The mouse anti-HLA class I (HLA class-A, B, C) mAb (clone: W6/32) was purchased from Harlan Laboratories Inc. (IN). Alexa Flour^®^ 488-conjugated goat anti-mouse IgG was purchased from Invitrogen (CA). Mouse IgG was purchased from Abcam (Cambridge UK). The mAbs for WT-1 (clone: 6F-H2), Calretinin (clone: DAK-Calret 1), Podoplanin (clone: D2-40), Cytokeratin 5/6 (clone: D5/16 B4), EMA (clone: E29), Carcinoembryonic Antigen (CEA) (clone: II-7), TTF-1 (clone: 8G7G3/1), and Epithelial-Related Antigen (clone: Moc-31) were purchased from DAKO. The mAb against Mesothelin (clone: 22A31) and anti-GLUT-1 polyclonal Ab were purchased from IBL (Japan). Anti-CD26 mAbs (clone 1F7, 5F8) were established in our laboratory [25].

Anti-CD25 mAb (clone: BC96) and mouse Ig G1 isotype control (MOPC-21) were purchased from TONBO biosciences (CA).

### Generation of mAbs against mesothelioma cells

An eight-week-old female BALB/c mouse was immunized alternately using intraperitoneal injections of 2–5 × 10^6^ living cells derived from the mesothelioma cell lines ACC-MESO4, MSTO-211H, and NCI-H226 every 2 weeks for 3 months. Three days after the last immunization, spleen cells were fused with P3U1-nonproducing myeloma cells using polyethylene glycol 4000 (Merck, Darmstadt, Germany) and cultured in RPMI-1640 supplemented with 10 % fetal calf serum (FCS, Japan Bioserum, Fukuyama, Japan), 5 % BriClone (NICB, Dublin, Ireland), and HAT (Invitrogen, Tokyo, Japan) in the wells of 96-well flat-bottom plates (Costar, Corning Incorporated, Corning, NY). Hybridoma supernatants were screened for reactivity with the mesothelioma NCI-H2452 cells, which were not used for immunization. Light microscopy revealed morphological changes and an aggregation of target cells induced by incubation with supernatants of hybridoma clones after 72 h. The reactivity of hybridoma supernatants for the other mesothelioma cell lines was also determined. Four hybridomas, JMAM1–4, were selected and cloned using limiting dilution. The isotype of these mAbs was IgG1, and they cross-reacted widely with mesothelioma cell lines.

### Flow cytometric (FACS) analysis

The expression of the molecular target(s) of the newly established mouse anti-human mesothelioma mAbs was determined using a BD LSRForessa™ cell analyzer (BD Bioscience, CA, USA). Briefly, the human mesothelioma cell lines and the cell lines derived from other tumors were incubated with the supernatants of hybridomas and then with Alexa Flour^®^ 488-conjugated rat anti-mouse IgG (BD Bioscience) on ice.

### Inhibition test

NCI-H226 cells were first incubated with already known existing Abs and further incubated with Alexa Flour^®^ 488-labeled JMAM mAbs.

### Immunocytochemistry

Samples of pleural effusions submitted to the Department of Pathology, Juntendo University, were used for immunohistochemistry and other histological staining procedures. Three representative cases of cytologically diagnosed mesothelioma were used for immunohistochemistry, and all cases were stained with positive and negative antibody panels of diagnostic antibodies to confirm the diagnosis of mesothelioma. Cell smears were fixed in 95 % ethanol for Papanicolaou and immunostaining, or air-dried for May-Grunwald-Giemsa staining. Cell sediments were fixed with ethanol and embedded in paraffin for immunostaining. Sections were deparaffinized using three changes of xylene and rehydrated with a graded series of ethanol concentrations. Endogenous peroxidase was inactivated with 0.3 % H_2_O_2_ in phosphate-buffered saline for 10 min. Samples were incubated with the novel anti-mesothelioma mAbs JMAM1−4 at 4 °C overnight in a humidified chamber, followed by the addition of EnVision™ + DualLink (DAKO) and 3.3′-diaminobenzidine (Dojindo Laboratories) as the chromogen. Cell smears and cell-block sections were counterstained to reveal nuclei using Mayer’s hematoxylin. For all cases, we stained cell block sections using antibodies against EMA, Podoplanin, GLUT-1, Calretinin, WT-1, Cytokeratin 5/6, Mesothelin, CEA, TTF-1, and epithelial-related antigen to confirm the diagnosis of mesothelioma (Table 1).

**Table 1:** Antibodies used in this study and immunocytochemical reactivity

Antibody	Clone	Source	Dilution	Specimen	Case 1	Case 2	Case 3
JMAM1		Our labolatory	Undiluted cell supernatant	Cell smear	+	+	+
JMAM2		Our labolatory	Undiluted cell supernatant	Cell smear	+	+	+
JMAM3		Our labolatory	Undiluted cell supernatant	Cell smear	+	+	+
JMAM4		Our labolatory	Undiluted cell supernatant	Cell smear	+	+	+
WT-1	6F-H2	DAKO	1:200	Cell block	+	±	+
Carletnin	Dak-Calret 1	DAKO	1:100	Cell block	+	+	+
Mesothelin	22A31	IBL	1:1000	Cell block	+	+	+
Podoplanin	D2–40	DAKO	1:200	Cell block	+	+	+
CK5/6	D5/16B4	DAKO	1:50	Cell block	+	+	+
EMA	E29	DAKO	1:100	Cell block	+	+	+
GLUT-1	5B12.3	IBL	1:1000	Cell block	+	+	+
CEA	II-7	DAKO	1:50	Cell block	−	−	−
TTF-1	8G7G3/1	DAKO	1:100	Cell block	−	−	−
Epithelial-related antigen	Moc-31	DAKO	1:100	Cell block	−	−	−

### In vitro proliferative assay of mesothelial cells incubated with mAbs

MSTO-211H cells (1 × 10^4^ cells/well) were incubated with 10 % FCS-RPMI supplemented with 0.005–0.4 µg/ml of JMAM1–4 mAbs for 48 h at 37 °C in an atmosphere containing 5 % CO_2_. The culture was pulsed with 0.5 μCi of tritiated thymidine, [^3^H]-TdR, for the final 24 h. The incorporation of [^3^H]-TdR was determined using scintillation counting. Data are expressed as the mean ± standard deviation (SD) of triplicate samples and represent three separate experiments.

### Wound-healing assay

Mesothelioma NCI-H226 cells were seeded in 6-well plates (Corning) and grown to 90 % confluence in RPMI plus 10 % FCS. The cell monolayer was wounded with the tip of an Eppendorf P200 pipette. After wounding, the wells were washed with media to remove dead cells and debris. The wells were treated with either 3 µg/ml of anti-mesothelioma mAbs (JMAM1–4) or a control IgG (3 µg/ml) and cultured further. The wound closure was observed after 24 h.

### Cell invasion assay

For the cell invasion test, a Corning Matrigel Invasion Chamber (8-μm pore size, coated with Matrigel; Discovery Labware Inc., Bedford, MA, USA) was placed into the wells of 24-well culture plates; RPMI-1640 medium with 10 % serum and JMAM1–4 mAbs (each 10 μM) were added into the lower chamber; then, 2 × 10^4^ NCI-H226 cells in serum-free RPMI-1640 medium was added to the upper chamber and cultured at 37 °C. After 15 h of incubation, the cells on the upper surface of the filter membrane that had not migrated were gently scraped away with a cotton swab. The invading cells on the lower surface of the filter membrane were fixed with methanol, stained with Diff-Quick™ (Sysmex), and counted as described above. All tests were performed in triplicate.

### Statistical analysis

The data were analyzed using Student’s *t* test. The results are expressed as the mean ± SD and P values of <0.05 were considered significant. Statistical analyses were performed using SPSS 14.0 software (IBM, NY).

## Results

### Morphological changes of mesothelioma cell lines induced by the newly generated mAbs

**Fig. 1:**
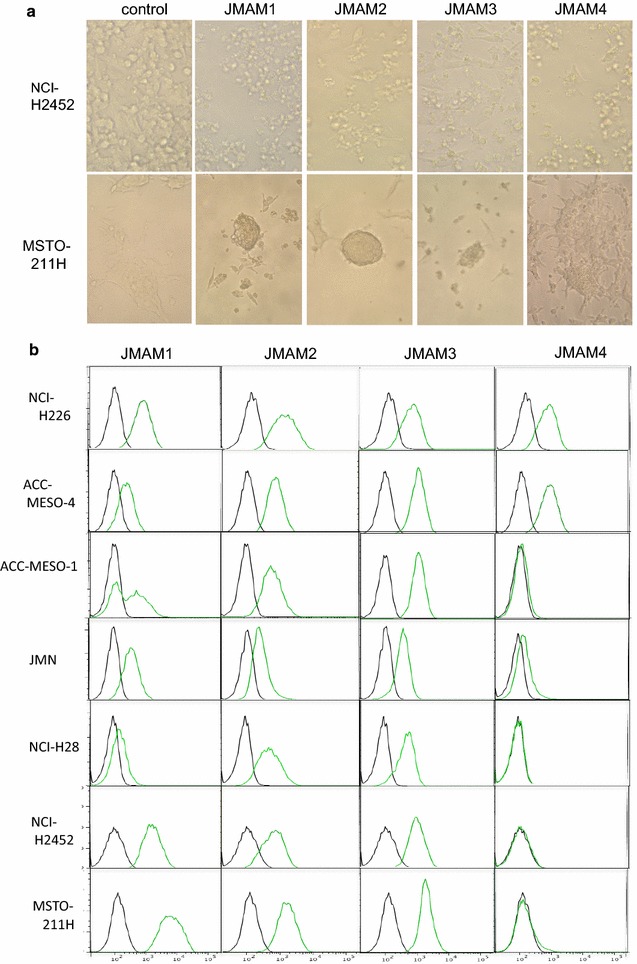
Reactivity of JMAM mAbs with mesothelioma cell lines. **a** Morphological changes by JMAM mAbs. NCI-H2452 cells were incubated with hybridoma supernatants for 72 h and observed using visible light microscopy. RPMI-1640 medium with 10 % FCS served as the control (*upper column*). These findings were also reproduced using MSTO-211H cells (*lower column*). **b** Flow cytometry analysis of JMAM mAb reactivity. Mesothelioma cell lines were incubated with the hybridoma supernatant (*green* histogram) or control mouse IgG (*black* histogram), subsequently stained with Alexa Flour^®^-488 labeled anti-mouse IgG Ab and analyzed using flow cytometry

We found that the newly generated four mAbs reproducibly induced morphological changes in a mesothelioma cell line that was not used for immunization. Light microscopy revealed that the morphology of the NCI-H2452 cells changed from spindle-shaped to round, and the numbers of these cells decreased after incubation with JMAM1–4 mAbs for 72 h compared with control mouse IgG (Fig. 1a, upper column). These morphological changes indicated that the mAbs bound the mesothelial cell lines. These findings were also reproduced using MSTO-211H cells that were used for immunization (Fig. 1a, lower column). Furthermore, these mAbs aggregated MSTO-211H cells. Taken together, these findings indicate that the newly established mAbs reacted with the mesothelial cell lines.

### Analysis of the binding of mAbs to the mesothelial cell lines

The reactivity of the mAbs against the mesothelial cell lines was determined using FACS analysis. JMAM1, JMAM2 and JMAM3 mAbs stained the epithelial (ACC-MESO-4, JMN) and sarcomatous (MSTO-211H, H2452, H28 and MESO-1) cell lines. In contrast, JMAM4 stained the epithelial cell lines but not the sarcomatous cell lines (Fig. 1b).

### Competitive inhibition of JMAM mAbs with established mAbs

To determine whether the newly established JMAM mAbs bind to the same epitope of the already existing Abs, we performed an inhibition test by flow cytometry.

**Fig. 2:**
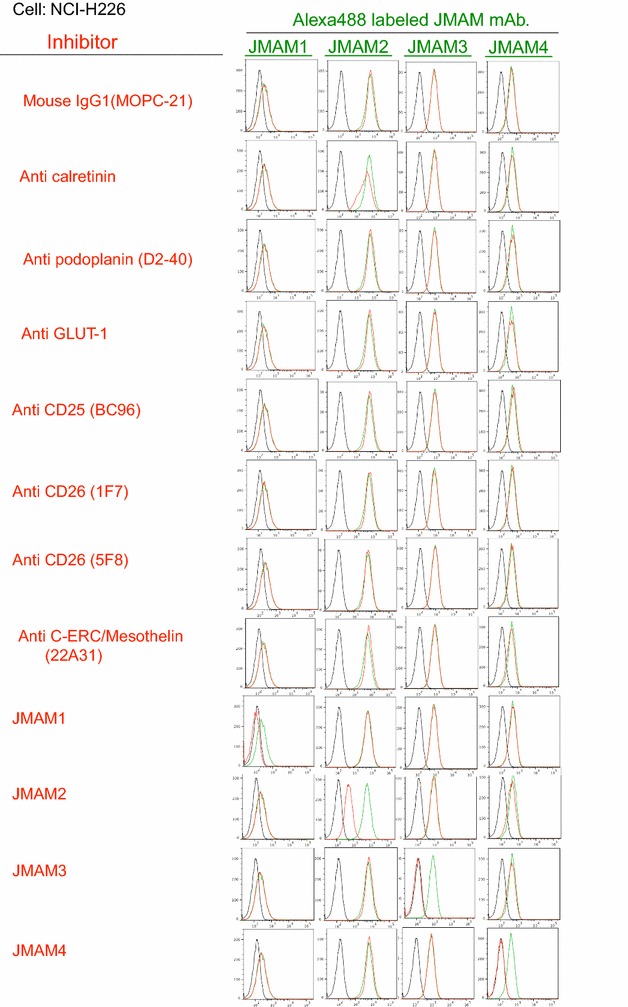
Competitive inhibition of JMAM mAbs with established mAbs. Staining profiles of JMAM mAbs without or with already existent mAbs are shown by *green* *lines* or *red* *lines*, respectively

NCI-H226 cells were incubated with JMAM mAbs followed by staining with existing Abs already known to bind to mesothelioma [anti-calretinin, anti-podoplanin (D2-40), anti-GLUT-1, anti-CD25 (BC96), anti-CD26 (1F7, 5F8), anti-C-ERC/mesothelin (22A31)]. (Fig. 2).

Anti-calretinin was able to partially inhibit the staining of the NCI-H226 mesothelioma cell line with JMAM2 mAb. This result indicates that the JMAM2 determinant is strongly related to calretinin; however, the other JMAM mAbs have no relationship with already existing Abs, they may bind to mesothelioma cells.

### Analysis of the binding of mAbs to other tumor cell lines

**Fig. 3:**
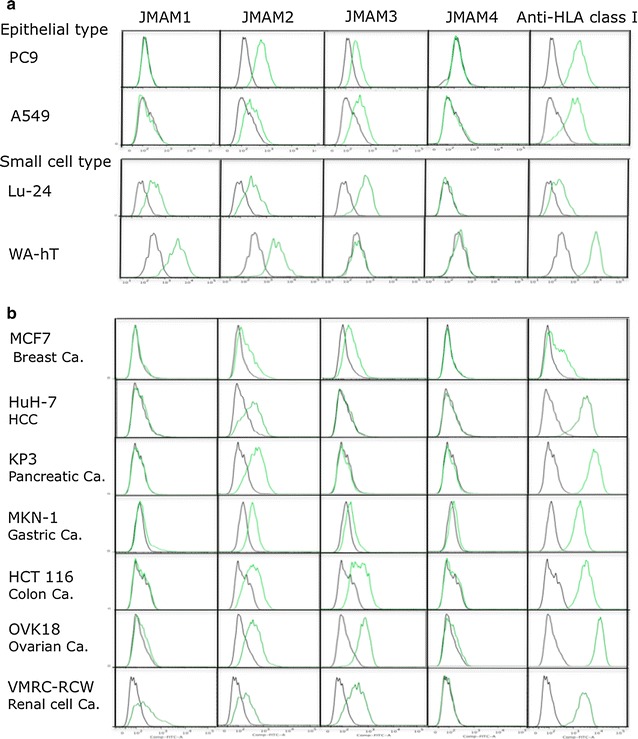
Reactivity of JMAM mAbs with other cell lines. Epithelial-type and small-cell-type lung cancer (**a**) and other cancer cell lines (**b**) were incubated with hybridoma supernatants (*green* histogram) or control mouse IgG (*black* histogram), subsequently stained with Alexa Flour^®^ 488-labeled anti-mouse IgG Ab and analyzed using flow cytometry

Using FACS analysis, we next determined whether the mAbs bound to lung cancer cell lines. Binding of JMAM1 to epithelial-type lung cancer cell lines (A549 and PC9) was not detectable. In contrast, it bound to the small-cell lung cancer cell lines WA-hT and Lu24. The extent of binding of JMAM2 and JMAM3 mAbs to lung cancer cell lines varied. The JMAM4 mAb did not bind to any of the lung cancer cell lines (Fig. 3a).

To determine the cross-reactivity of these novel anti-mesothelioma mAbs to cell lines derived from tumors other than those of the lung, we used FACS analysis to determine their ability to react with MCF7 (breast cancer), HuH-7 (liver cancer), KP3 (pancreatic cancer), MKN-1 (gastric cancer), HCT 116 (colon cancer), OVK18 (ovarian cancer), and VMRC-RCW (renal cell carcinoma) cell lines. The JMAM1 mAb only cross reacted with the VMRC-RCW cell line. JMAM4 mAbs did not react detectably with any of these carcinoma cell lines. The JMAM2 mAb slightly or significantly stained all carcinoma cell lines tested. The JMAM3 mAb did not stain the liver or pancreatic cancer cell lines; however, it lightly stained a gastric cancer cell line and strongly stained breast and colon cancer cell lines (Fig. 3b). Taken together, these data indicate that the JMAM1 mAb distinguished mesothelioma and small-cell lung cancer cells from epithelial lung cancer cells as well as any other cancer cells derived from these tissues except for renal cell carcinoma. These data also suggesting that JMAM4 mAb may distinguish epithelial mesotheliomas from all other cancers.

### Analysis of mesotheliomas by mAbs

To determine whether these newly generated mAbs were suitable for immunohistochemical staining of formalin-fixed tissue sections, we used them to analyze surgically resected mesothelioma tissue specimens. Unfortunately, we did not detect staining of formalin-fixed paraffin-embedded tissue specimens (data not shown). The antigens recognized by the mAbs might have been masked or antigenically inactivated by formalin fixation.

**Fig. 4:**
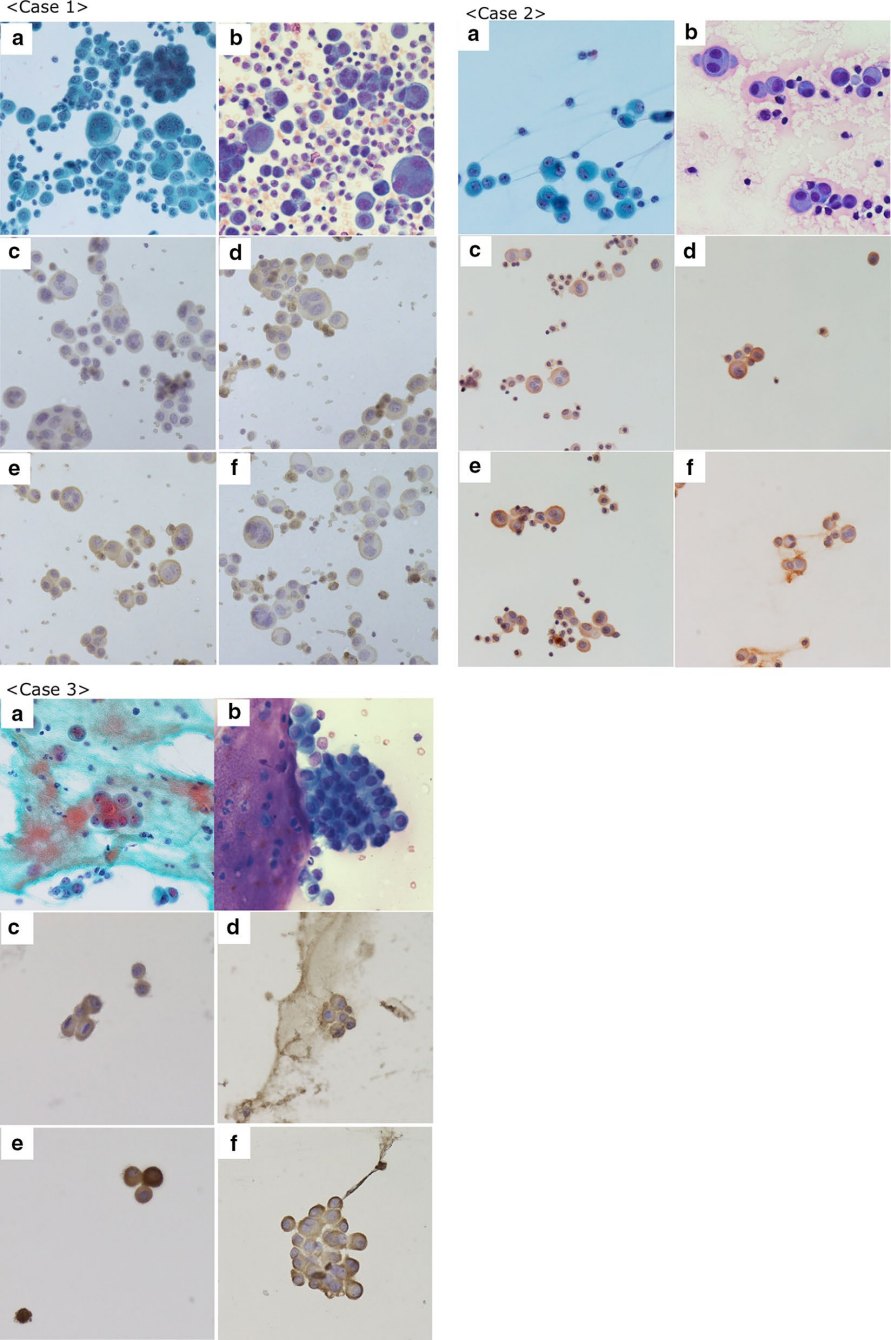
Immunocytochemistry of clinical cases. **a** Papanicolaou; **b** Giemsa; **c** JMAM1; **d** JMAM2; **e** JMAM3; **f** JMAM4. Original magnification 400×. The malignant mesothelioma smear specimens, prepared from pleural effusions. *Case 1* JMAM1, JMAM2, JMAM3, and JMAM4 mAbs stained membranes. JMAM2 mAb stained membranes and the cytoplasm. *Case 2* All mAbs stained membranes. *Case 3* JMAM1 and JMAM4 mAbs stained membranes; JMAM2 and JMAM3 mAbs stained membranes and cytoplasm

Body fluid retention is one of the symptoms of many patients with malignant mesothelioma who are often diagnosed with mesothelioma by body fluid cytological examination. Therefore, we investigated whether the mAbs reacted with cytological samples using body fluid specimens that were fixed with ethanol. We analyzed specimens from three patients with malignant mesothelioma and those from patients suspected to have malignant mesothelioma (Fig. 4). All materials were prepared from pleural effusions.

*Case 1* Many cell clusters were present, including tight and loose clusters with flattened cellular borders. Individual cells showed wide variation in shape and size, ranging from small to very large. The JMAM1–4 mAbs stained membranes, whereas the JMAM2 mAb stained the cytoplasm and the membrane.

*Case 2* The few malignant cells present were judged Class III by Papanicolaou classification. All mAbs stained membranes clearly.

*Case 3* Present were small to large clusters with knobby borders and a single-cell population. These cells had low nuclear: cytoplasmic ratios, but occasionally showed macronucleoli. The staining of membranes by mAbs JMAM1–4 was distinct. Antibodies against Podoplanin, Mesothelin, EMA, and GLUT-1 stained cell-block specimens, which confirmed these atypical cells as derived from mesothelioma (Table 1). Because JMAM1–4 mAbs stained the membranes of all mesothelioma specimens tested, they may be useful for the cytological testing of pleural effusions of patients with mesothelioma.

### Histopathology and cytology of lung and mesothelial cells

**Fig. 5:**
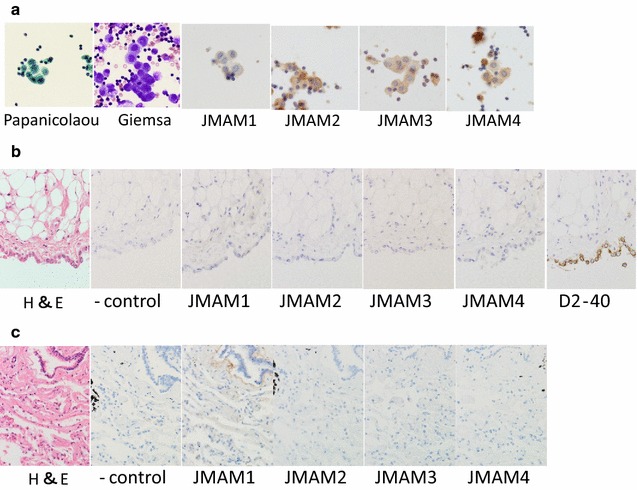
Histopathology and cytological findings of reactive mesothelial cells, normal pleura and lung, with immunoreactive features. **a** Pleural effusion (reactive mesothelial cells, 1000×). **b** Normal pleura (400×). **c** Normal lung (400×)

Reactive normal pleura and lung immunoreactive features are shown in Fig. 5. Reactive pleural effusion mesothelial cells stained with JMAM mAbs (a); however, normal pleural mesothelium and lung tissue did not stain with JMAM mAbs (b, c).

### Analysis of the effects of mAbs on the proliferation of mesothelioma cells

**Fig. 6:**
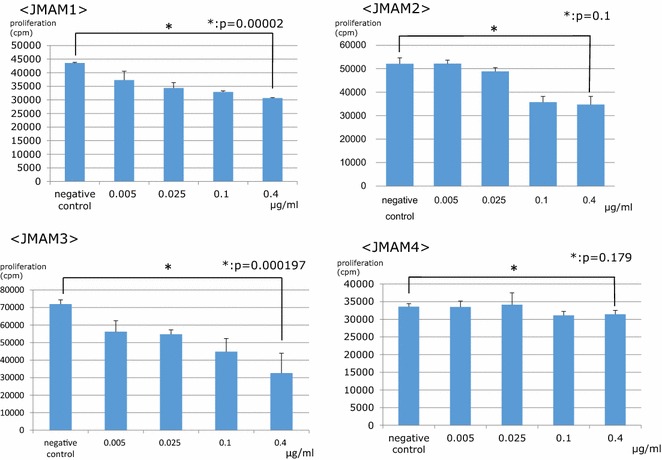
Proliferation of MSTO-211H cells in the presence of JMAM1–4 mAbs. JMAM1–4 mAbs were added at the indicated concentrations to cultures of MSTO-211H mesothelioma cells. JMAM1, P < 0.05; JMAM2, P = 0.1; JMAM3, P < 0.05; JMAM4, P = 0.18

We next tested whether the mAbs inhibited the proliferation of the MSTO-211H mesothelioma cell line (Fig. 6). The JMAM1 and JMAM3 mAbs inhibited the proliferation of MSTO-211H cells as a function of their dose. Thus, proliferation was reduced by at least 50 and 40 % by 0.4 μg/ml of JMAM1 or JMAM3 mAbs, respectively. The JMAM2 mAb inhibited cell proliferation to some extent. The JMAM4 mAb did not inhibit the proliferation of MSTO-211H cells. These results indicate that JMAM1 and JMAM2 mAbs may be useful for treating patients with mesothelioma, at least those with the epithelioid phenotype.

### Analysis of the effect of JMAM mAbs on migration of mesothelioma cells

**Fig. 7:**
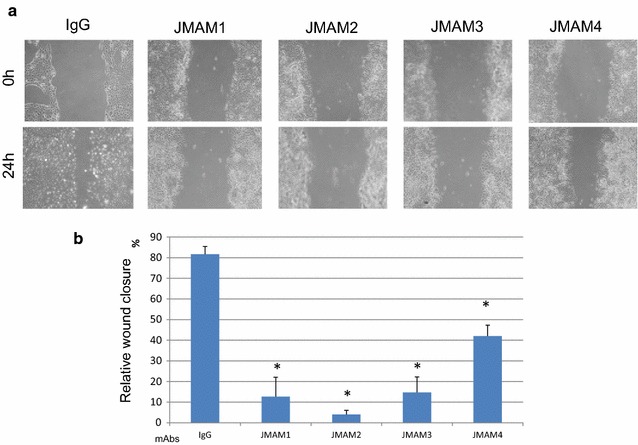
Wound-healing assays. **a** Representative images of wound closure assays after NCI-H226 cells were incubated with anti-mesothelioma antibodies or control mouse IgG. **b** The extent of wound closure was calculated by analyzing the scratched area covered by the cells after 24 h using ImageJ software. The data were normalized to the control values. The data are represented as the mean ± SD of three independent experiments. *P < 0.05; treated versus IgG control

Cell motility is an essential process of tumor metastasis and progression. Therefore, we investigated whether the mAbs would affect the migration of mesothelioma cells using a wound-healing assay. The anti-mesothelioma mAbs JMAM1, JMAM2, and JMAM3 significantly inhibited the ability of NCI-H226 cells to migrate to and close an experimentally induced wound (Fig. 7). These results show that the anti-mesothelioma mAbs inhibited the motility of mesothelioma cells and suggest that the JMAM1–4 mAbs may possess a remarkable ability to inhibit the progression of mesotheliomas.

### Analysis of the effect of JMAM mAbs on invasion of mesothelioma cells

**Fig. 8:**
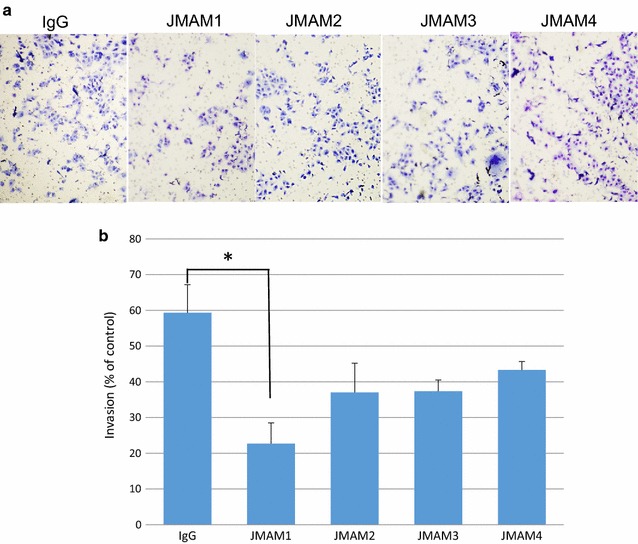
Analysis of the effect of JMAM mAbs on invasion of mesothelioma cells. Cells (NCI-H226) were seeded into the *upper chamber* and the *lower chamber* was filled with medium as described in the “Methods” section. Photos were captured by a light microscope at ×200 (**a**). The migration of cells was measured by counting the migrating cells on the lower surface of the membrane in 3 fields (**b**). JMAM1, *P < 0.05; JMAM2, P = 0.12; JMAM3, P = 0.08; JMAM4, P = 0.18. Each mAb treated versus IgG control

We assessed the cell invasion ability of mesothelioma NCI-H226 cells. Significant inhibition of invasion was observed by JMAM mAbs compared with control mouse IgG1 Ab (Fig. 8). JMAM1 mAb significantly decreased the trans membrane migration of NCI-H226 cells compared with cells treated with the mouse IgG1 isotype control.

## Discussion

Differentiating between a mesothelioma and a papillary adenocarcinoma is sometimes very difficult [25]. Clinicians recommend that the definitive diagnosis of mesothelioma may be achieved using immunohistochemical analysis of cytological or histological specimens with currently available antibodies [8]. Many mAbs are developed for the diagnosis of and therapy for mesothelioma. Previously, we focused on CD26 as a novel therapeutic target for mesothelioma and have developed a humanized anti-CD26 mAb (clone: YS110), which is currently being evaluated in a phase I clinical trial for patients with malignant mesothelioma [26]. We also developed anti-human CD26 mAbs (clone: 1F7, 5F8) that clearly and reliably detect the denatured CD26 molecule in formalin-fixed paraffin-embedded tissue [27]. Although many antibodies are available to aid diagnosis, no single antibody can unambiguously distinguish mesotheliomas from other carcinomas. Anti-CD26 mAb reacts with epithelial and sarcomatoid mesotheliomas. However, anti-CD26 mAb also reacts with several other tumor cells and lymphocytes. Anti-mesothelin mAbs react with epithelial but not sarcomatoid mesotheliomas [13]. In contrast, JMAM1, JMAM2, and JMAM3 mAbs react with both subtypes. Anti-mesothelin mAbs react with renal cell carcinomas and pancreatic and ovarian cancers [28–30], unlike the JMAM4 mAbs that did not react with lung, ovarian, or renal cell carcinomas or any of the other cancer cell lines tested. JMAM1 mAb did not cross-react with other carcinoma cell lines except for small-cell-type lung cancer cell lines and renal cell carcinoma cell lines. Taken together, our newly generated mAbs are more specific for mesothelioma than other diagnostic mAbs and may be helpful in the differential diagnosis of mesothelioma. Novel anti-mesothelioma mAbs may either serve as tools to diagnose mesotheliomas as stand-alone reagents or together with other diagnostic mAbs.

It is now commonly accepted that therapeutic mAbs dramatically improve the treatment of cancer patients. However, after repeated therapy, not all patients respond to therapeutic mAbs [31] because clones appear during the course of treatment those do not express the target. Therefore, additional therapeutic options are required to treat these patients. Common therapeutic mAbs against cell surface molecules exert their effects largely through immunological mechanisms, including complement-dependent cytotoxicity (CDC) and antibody-dependent cellular cytotoxicity (ADCC). ADCC and CDC may not be effective for treating patients with cancer because the patients may be immunocompromised due to radiation, chemotherapy, and the malignancy itself. However, in addition to indirectly inducing Fc-dependent cell death, several mAbs possess a direct antitumor effect that induces cell arrest or programmed cell death [32, 33].

Therefore, in this study, we investigated reactivity with mesothelioma cells as well as the direct antitumor effect of the newly generated anti-mesothelioma mAbs. We found that JMAM1 and JMAM3 mAbs inhibited the proliferation of the MSTO-211H mesothelial cell line, and JMAM1–4 mAbs inhibited wound closure by NCI-H226 cells to varying degrees. JMAM1–4 also inhibited invasion of NCI-H226 cells to various degrees. Unfortunately, we did not identify the target molecules of JMAM1–4 mAbs. Nevertheless, the promising anti-mesothelioma activities of these antibodies warrant continued studies in vitro and in vivo that will include efforts to identify their targets. Our strategy for generating diagnostic and therapeutic mAbs specific for tumor cells differs from those of conventional methods that employ immunization with peptides or DNA. Specifically, we immunized a mouse with three different mesothelioma cell lines and screened the antibodies using a different mesothelioma cell line to potentially obtain novel mAbs that react with unknown targets on the surface of mesothelioma cells.

## Conclusion

Newly established anti-mesothelioma mAbs, JMAM1–4, are potentially useful as diagnostic and therapeutic agents for mesothelioma. Moreover, our novel strategy for establishing anti-tumor mAbs may facilitate the development of new diagnostic and therapeutic techniques for other malignancies as well as mesothelioma.
